# Supplementary material for: Systematic assessment of commercially available low-input miRNA library preparation kits
Source: RNA Biol. 2019 Sep 27;17(1):75–86. doi: 10.1080/15476286.2019.1667741 (PMC6948978; doi:10.1080/15476286.2019.1667741)
Supplement: Supplemental Material [file krnb-17-01-1667741-s001.zip › Supplementary information/Supplementary_figure_S2.pdf]

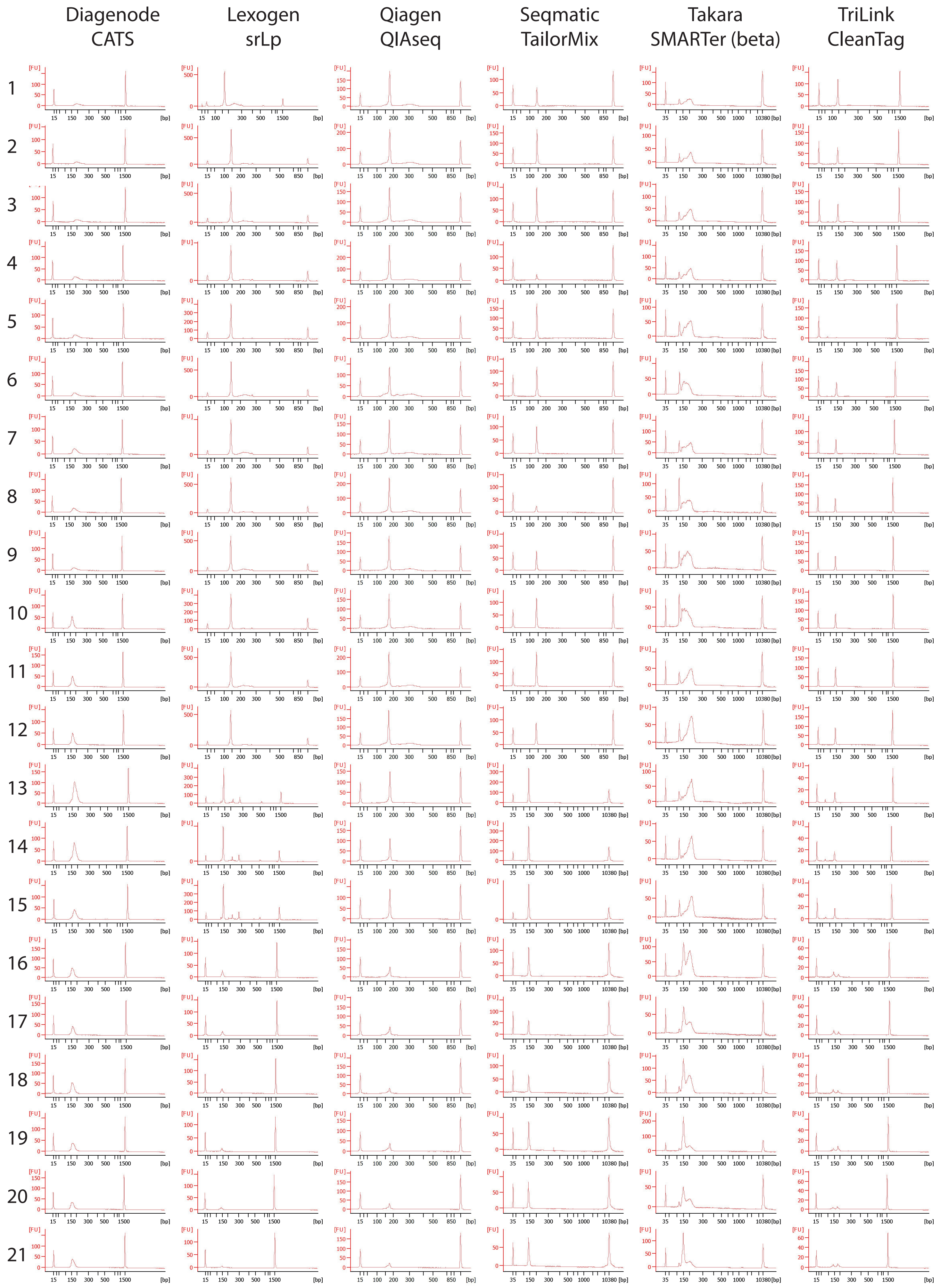
Diagenode CATS
Lexogen srLp
Qiagen QIAseq
Seqmatic TailorMix
Takara SMARTer (beta)
TriLink CleanTag
1
2
3
4
5
6
7
8
9
10
11
12
13
14
15
16
17
18
19
20
21
[FU]
[bp]
